# Endoscopic Surgery for Supratentorial Deep Cavernous Malformation Adjacent to Cortical Spinal Tract: Preliminary Experience and Technical Note

**DOI:** 10.3389/fneur.2021.678413

**Published:** 2021-06-21

**Authors:** Fuxin Lin, Chunwang Li, Xiaorong Yan, Dengliang Wang, Yuanxiang Lin, Dezhi Kang, Changzhen Jiang

**Affiliations:** ^1^Department of Neurosurgery, Neurosurgery Research Institute, The First Affiliated Hospital, Fujian Medical University, Fuzhou, China; ^2^Clinical Research and Translation Center, The First Affiliated Hospital, Fujian Medical University, Fuzhou, China; ^3^Fujian Key Laboratory of Precision Medicine for Cancer, The First Affiliated Hospital, Fujian Medical University, Fuzhou, China; ^4^Key Laboratory of Radiation Biology of Fujian Higher Education Institutions, The First Affiliated Hospital, Fujian Medical University, Fuzhou, China

**Keywords:** cerebral cavernous malformations, endoscopic surgery, cortical spinal tract, DTI navigation, technical note

## Abstract

In this study, we aimed to introduce a technical note and to explore the efficacy of endoscopic surgery combined with diffusion tensor imaging (DTI) navigation for supratentorial deep cerebral cavernous malformations (CCM). A prospectively maintained database of CCM patients was reviewed to identify all CCM patients treated by endoscopic surgery. The sagittal T1-weighted anatomical magnetic resonance imaging (MRI) and DTI were acquired before surgery. Endoscopic surgery was planned and performed based on preoperative DTI images and intraoperative DTI navigation. All patients were followed up more than 6 months. Motor function deficit and modified Rankin scale (mRS) scores were documented on follow-up. A final mRS score of 0–2 was considered a good outcome, and a final mRS score >2 was considered a poor outcome. Second DTI and 3DT1 were performed at 3 months after surgery. We tracked the ipsilateral corticospinal tract (CST) on pre- and postoperative DTI images. The overall mean FA values and the number of fibers of tracked CST were compared on pre- and postoperative DTI images. Risk factors associated with motor deficits and poor outcomes were analyzed. Seven patients with deep CCM and treated by endoscopic surgery were enrolled in this study. The mean value of preoperative mRS was 1.5 ± 0.98, but that score recovered to 0.86 ± 1.22 3 months later. The mRS scores were improved significantly according to statistical analysis (*p* = 0.012). According to the Spearman non-parametric test, only the fiber number of ipsilateral CST on postoperative DTI was significantly associated with muscle strength 6 months after surgery (*p* = 0.032). Compared with preoperative CST characteristics on DTI, the change of FA value (*p* = 0.289) and fiber number (*p* = 0.289) of ipsilateral CST on postoperative DTI was not significant It meant that the CST was protected during endoscopic surgery. Endoscopic surgery based on DTI navigation might be an effective method to protect fiber tracts in supratentorial deep CCM patients and improve long-term outcomes. However, more studies and cases are needed to confirm our findings.

## Introduction

Deep cerebral cavernous malformations (CCM) account for 9–35% of intracranial CCMs, including corona radiata, paraventricular, intraventricular, insula, basal ganglia, and brainstem CCMs (1). For patients with rebleeding history, enlarged lesions, drug-refractory epilepsy, and progressed functional deficits, surgical treatment is needed. Surgical resection is often performed under a microscope with the help of a plate retractor. However, deep location, the long corridor of the surgical approach, distorted anatomical structures, and the indeterminate location of the fiber tracts make microscopic surgical resection challenging. The morbidity is about 10–30%, and the mortality is about 1.9% after microscopic surgical resection for deep CCMs (1, 2). Accurate positioning and sparing adjacent eloquent fibers are the key points for the safe resection of deep CCMs. With the rapid advancement of imaging software and transparent tube sheath, endoscopic surgery has gradually become an effective and well-accepted method for treating deep intracranial hemorrhage (ICH) and supratentorial parenchymal tumors (3, 4). However, its benefits have not been determined in deep CCM patients. Combining the accuracy of intraoperative neuronavigation and minimally invasive endoscopic surgery, it may be an effective way to remove supratentorial deep CCMs. In this study, we introduced the technical notes and reported the outcomes of endoscopic surgery patients, to determine the efficacy of endoscopic surgery combined with diffusion tensor imaging (DTI) navigation for supratentorial deep cavernous malformations.

## Methods

### Patients

A prospectively maintained database of CCM patients referred to The First Affiliated Hospital of Fujian Medical University was reviewed to identify all surgically treated CCM patients (ClinicalTrials.gov Identifier: NCT03467295). Patients were continuously enrolled at the interval between August 2018 and June 2020. The inclusion criteria were as follows: (1) supratentorial deep CCM including corona radiata, paraventricular, intraventricular, insula, basal ganglia, and thalamic; (2) patients had preoperative (1 week) and postoperative (3 months) DTI and treated by endoscopic surgery; (3) the distance between CCM and corticospinal tract (CST) was <1 cm on preoperative DTI images; (4) pathological diagnosis confirmed CCM; (5) follow-up at least 6 months; and (6) informed consent. The exclusion criteria were as follows: (1) accompanied with other cerebral vascular malformations, except developmental venous abnormalities (DVA); (2) acute intracranial hemorrhage within 1 week; and (3) intracranial tumors or previous history of brain trauma and surgery. The study was approved by the Institutional Review Board of The First Affiliated Hospital of Fujian Medical University. Written informed consent was obtained from all the patients or their guardians, when they were enrolled in our CCM database.

### Neuroimaging and Tractography

The details of neuroimages and tractography have been shown in our prior studies (5, 6). Conventional brain MRI and computed tomography (CT) scans were obtained as part of the diagnostic workups. The sagittal T1-weighted anatomical MR images (3DT1) and DTI were acquired using a 3.0-T MR system (SIEMENS Trio, Siemens Medical Solutions, Erlangen, Germany). The sagittal T1-weighted anatomical MR images were acquired using a gradient echo sequence with the following parameters: TR 2,300 ms, TE 2.98 ms, slice thickness 1 mm, 176 slices, FOV 256 mm, flip angle 9°, matrix 64 × 64, voxel size 1 × 1 × 1 mm, and a bandwidth of 240. The DTI studies were acquired using the diffusion-weighted echo-planar imaging technique with the following settings: TR 6,100 ms, TE 93 ms, slice thickness 3 mm, 45 slices, FOV 230 × 230 mm, matrix 128 × 128, and a motion-probing gradient in 30 orientations.

The generated image sets were processed on the iPlan 3.0 workstation (Brainlab, Feldkirchen, Germany). All image sets were automatically coregistered with each other and fused to the anatomical images using an automatic rigid registration. To track the CST, we used 2 regions of interest delineated in the precentral gyrus (seed) and peduncle where CST fibers were condensed (target). We selected a default fractional anisotropy threshold of 0.20 and a minimum fiber length of 70 mm using a deterministic algorithm. Two neuroradiologists, with consensus, documented the locations of the regions of interest and the tracked fibers. They were blinded to the clinical information of the patients. The processed preoperative datasets were then incorporated into the neuronavigation platform for intraoperative navigation.

### Endoscopic Surgery

**Figure 1:**
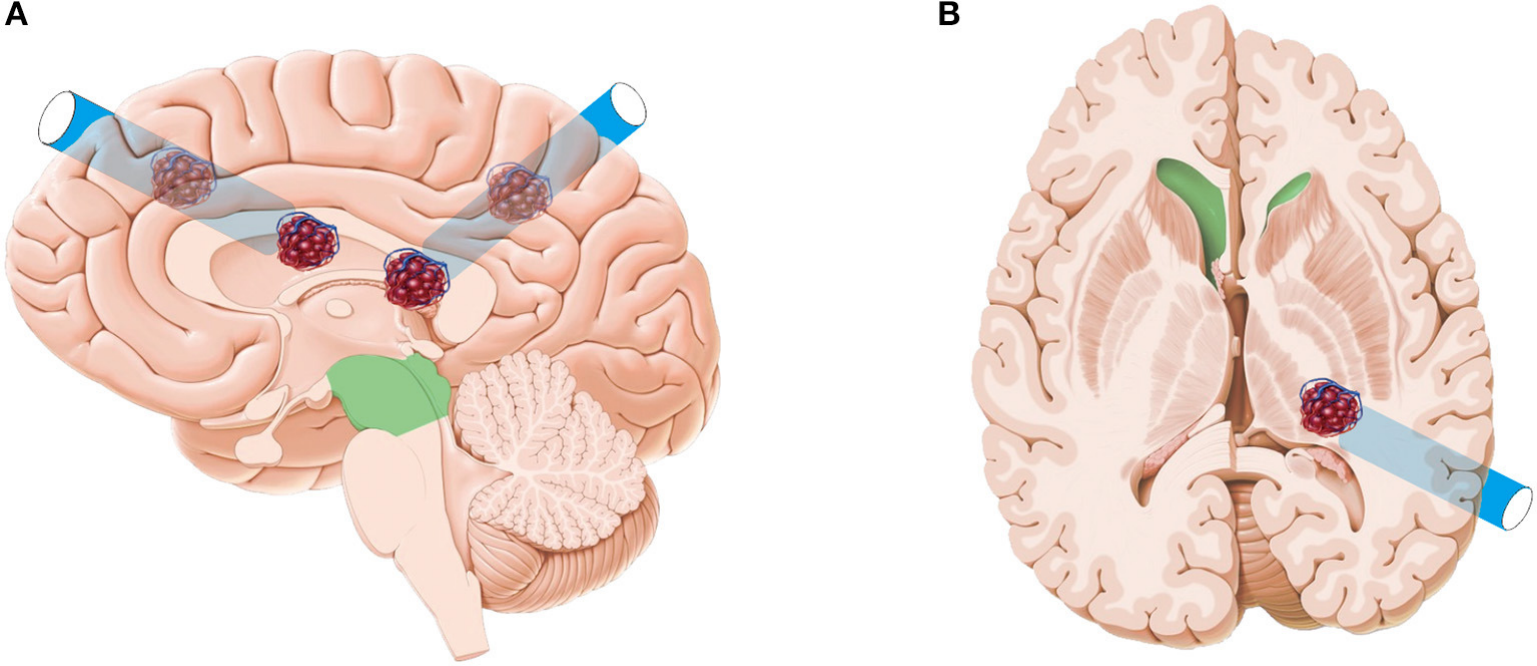
Artist's illustration depicting the frontal approach, paramedian parietal approach **(A)**, and parietooccipital approach **(B)**. The original pictures (brain anatomy and CCM) were from a website. This was designed by one of the authors with Adobe Photoshop CS5 and CorelDRAW 2019.

**Figure 2:**
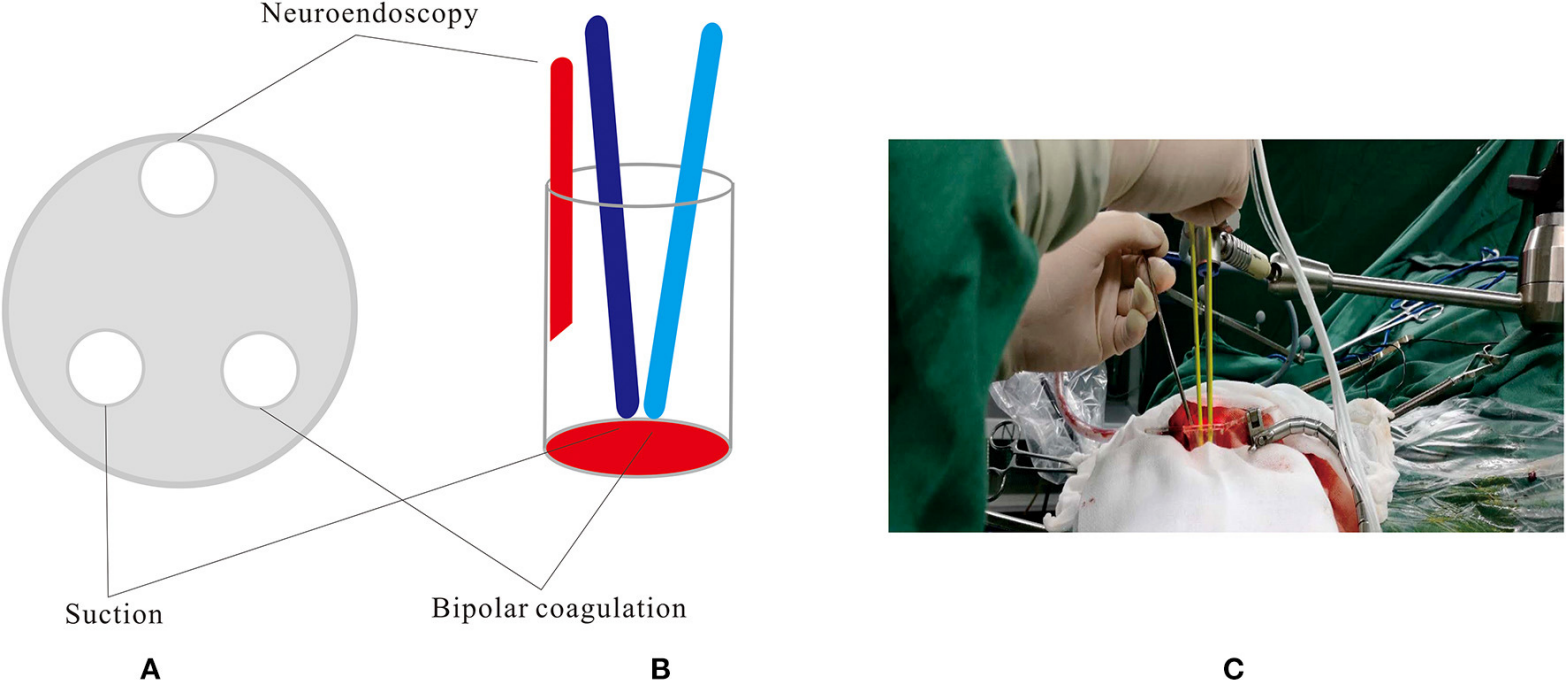
The rigid endoscopy was held by a fixed arm, while a suction and a bipolar coagulation were manipulated by the left and right hand, respectively. **(A,B)** Schematic diagram. **(C)** Intra-operative scene.

All endoscopic surgeries were conducted by the senior author CJ. A surgical corridor was planned before surgery based on preoperative DTI images, taking care to avoid traversing eloquent brain cortex and fiber tracts. A frontal approach was used for anterior deep CCMs, and a parietooccipital approach was used to treat lower basal ganglia and thalamic lesions. In cases involving posterior corona radiata and lateral ventricle, we commonly used a paramedian parietal approach (Figure 1). A 3–3.5-cm scalp incision and a 2.5–3-cm craniectomy were made. The dura was opened in a cruciate fashion. A small size coagulation and incision on the cortical pia were performed. A transparent plastic sheath—endoport (Levo Medical Technology Co., Ltd., Jilin Province, China)—was inserted stereotactically adjacent to the CCM guided by the pointer of neuronavigation. The model number of the endoport is LW211507 (70 × 15 × 21 mm). The rigid endoscopes used for surgery measure 0–45° and range between 2.7 and 4 mm in diameter (Karl Storz, Tuttlingen, Germany; Richard Wolf GmbH, Knittlingen, Germany). The rigid endoscopy was held by a fixed arm, while a suction and a bipolar coagulation were manipulated by the left and right hand, respectively (Figure 2). These instruments were brought alongside the endoscopy through the port. We dissected the lesion from the interface between the hemosiderin rim and the CCM. Associated venous anomalies and the hemosiderin rim were spared during surgery to protect adjacent fibers. The removed specimen was sent for pathological examination. Bleeding vessels, if seen, are irrigated or cauterized. Meticulous hemostasis is achieved and maintained under direct visualization. Conventional MRI was performed 1 month later to confirm the radical obliteration.

### Data Collection and Follow-Up

All data were prospectively collected using an electronic case report form through an electronic data capture (EDC) system (Real Data eClinical research system eRDDM, Real Data Medical Research Inc., Ningbo, China). Clinical data included demographic information (gender, age), initial presentation, physical examination [muscle strength of the upper and lower limbs, modified Rankin scale (mRS) score], and radiology information (Table 1). CCM location and CCM size (including the hematoma) were documented. All patients are assessed according to the reporting standards of cerebral CM research (7). Second DTI and 3DT1 were performed at 3 months after surgery. We tracked the pre- and postoperative CSTs. The overall mean fractional anisotropy (FA) values were obtained based on quantitative anisotropy on iPlan 3.0. The number of fibers of tracked ipsilateral CST was also recorded. The distance between the lesion and the fiber tract was also documented on sagittal T1-weighted anatomical images. Motor function deficit assessments were performed 7 days and at 6 months after surgery. All patients had a follow-up period that exceeded 6 months. The mRS score was also documented on follow-up. A final mRS score of 0–2 was considered as a good outcome, and a final mRS score >2 was considered as a poor outcome.

**Table 1:** Clinical characteristics and outcomes of the deep CCM patients treated by endoscopic surgery.

**No**.	**Gender**	**Age (years)**	**Location**	**Size (mm)**	**ULMS1**	**LLMS1**	**mRS1**	**CSTFA1**	**NCST1**	**ULMS2**	**LLMS2**	**mRS2**	**Follow-up (months)**	**ULMS3**	**LLMS3**	**mRS3**	**CSTFA2**	**NCST2**
1	Male	49	BGL	13.4	4	4	2	0.52	88	5	5	1	29	5	5	0	0.53	148
2	Female	50	BGL	27.8	3	4	3	0.43	159	1	4	3	27	3	4	3	0.4	24
3	Female	41	CRL	12.3	1	3	4	0.45	131	2	3	3	20	4	4	2	0.45	139
4	Female	44	CRL	37.6	4	5	2	0.46	1,257	5	5	1	26	5	5	0	0.48	1,802
5	Female	58	BGL	26.7	5	5	1	0.49	888	5	5	1	6	5	5	0	0.51	896
6	Male	44	BGL	10.7	4	5	2	0.35	112	5	5	1	7	5	5	0	0.38	295
7	Male	57	BGL	1.8	5	5	1	0.5	258	5	5	1	9	5	5	1	0.51	263

*BGL, CCMs located at the basal ganglia level; CRL, CCMs located at the corona radiata level; ULMS1, preoperative muscle strength of contralateral upper limb; ULMS2, muscle strength of contralateral upper limb at 7 days after surgery; ULMS3, muscle strength of contralateral upper limb at last follow-up; LLMS1, preoperative muscle strength of contralateral lower limb; LLMS2, muscle strength of contralateral lower limb at 7 days after surgery; LLMS3, muscle strength of contralateral lower limb at last follow-up; mRS1, preoperative modified Rankin score; mRS2, modified Rankin score at 7 days after surgery; mRS3, modified Rankin score at last follow-up; CSTFA1, the overall mean FA values of ipsilateral CST on preoperative DTI; CSTFA2, the overall mean FA values of ipsilateral CST on postoperative DTI; NCST1, the number of fibers of ipsilateral CST on preoperative DTI; NCST2, the number of fibers of ipsilateral CST on follow-up DTI*.

### Statistical Analysis

Data were reported as the mean and standard deviation (SD) for continuous variables or frequency for categorical variables. An independent-sample *t*-test or Spearman non-parametric test was used to determine the relationship between pre-/postoperative radiological features and motor deficits. Preoperative and final mRS scores were analyzed to identify the surgical outcomes in patients with deep CCM. Statistical tests were considered significant at *p* < 0.05. Preoperative CST features were compared with postoperative ones by paired-samples *t*-test to determine the intactness of the subcortical fibers. Statistical analysis was performed using the statistical package SPSS (version 20.0.0, IBM Corp., Armonk, NY, USA).

### Case Illustration

**Figure 3:**
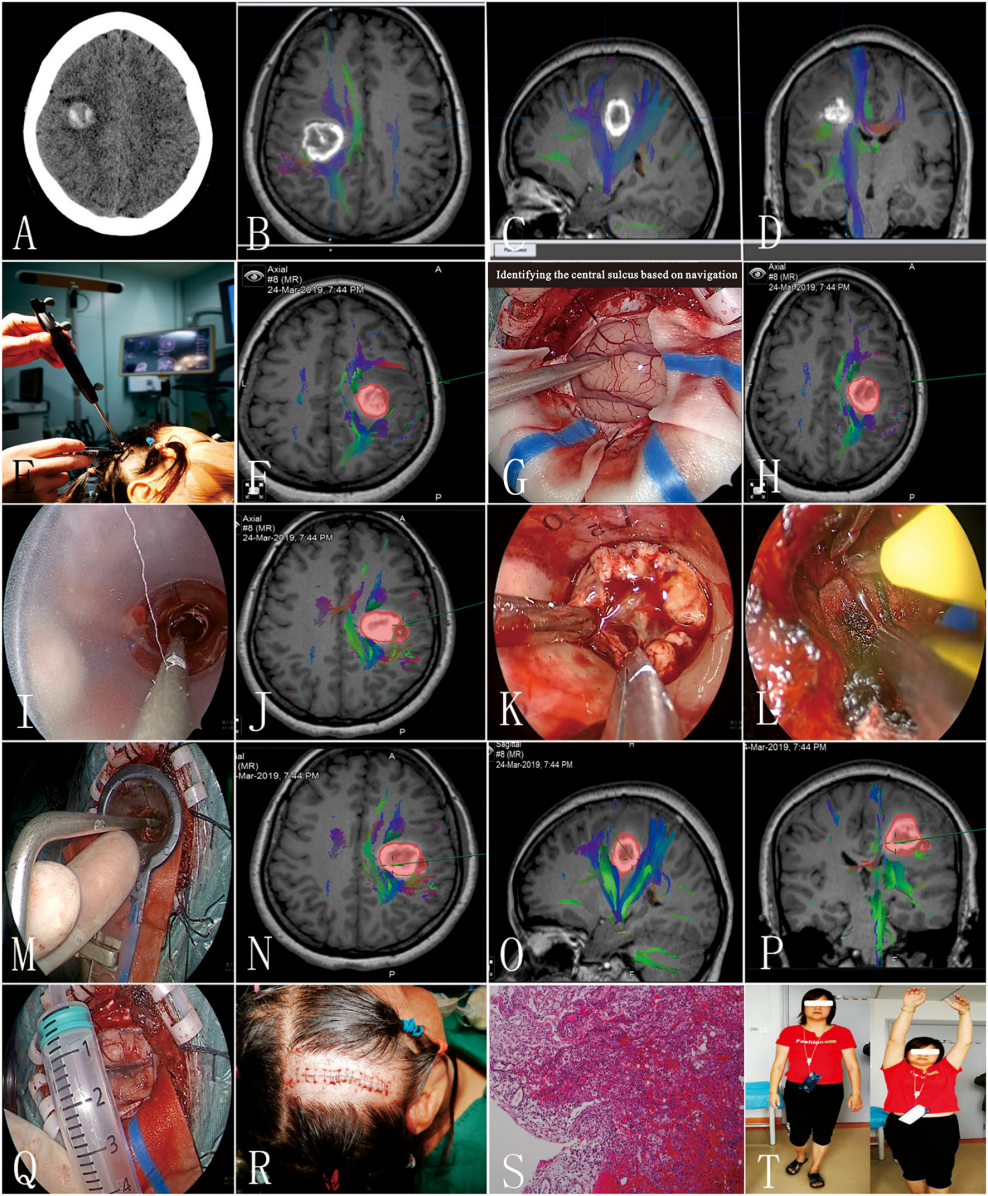
Clinical information of the typical patient (patient 3). **(A)** Preoperative CT scan; **(B–D)** preoperative diffusion tensor imaging (DTI) images; **(E,F)** preoperative navigation to design the incision; **(G,H)** identifying the central sulcus based on navigation; **(I,J)** elliptic endoport was inserted under the guidance of a navigation pointer; **(K)** the yellow-stained surface of the lesion; **(L)** homeostasis under direct vision; **(M–P)** identifying the medial border of the lesion; **(Q)** the width of the bone flap; **(R)** a small area shaving and a 5-cm linear incision were performed; **(S)** pathological results; **(T)** the patient visited the outpatient department 6 months after surgery.

A 41-year-old female patient presented with a sudden onset of paralysis and numbness of the left limbs 10 days ago. The muscle strength of the left upper and lower limbs was grade I and grade III, respectively. Hypotonia, ataxia, and decreasing of superficial sensibility of the left limbs were also detected. The Babinski sign was positive on the left side. Preoperative CT/MRI showed hematoma with mixed density located at the right frontal lobe on the corona radiata level (Figure 3A). The preliminary diagnosis was CCM bleeding. Preoperative DTI and 3DT1 showed that the lesion was just located under the central sulcus and involving the CST (Figures 3B–D). The mean FA value of the ipsilateral CST was 0.45, and the fiber number was 131. Considering the mass effect of hematoma, the motor and sensory function deficits, the risk of rebleeding from CCM, and the timing of resection, surgical treatment was recommended. The key points of surgery were as follows: (1) there is a need to protect the cortical and subcortical tissues to reduce surgical complications, (2) small lesion needs accurate positioning, and (3) radical resection is needed to avoid rebleeding. After multidisciplinary discussion, endoscopic surgery with intraoperative neuronavigation, and electrophysiological monitoring was scheduled. A small area shaving and a 5-cm linear incision were performed (Figures 3E,R). The diameter of the bone flap was 4 cm (Figure 3Q). Intraoperatively, after the lesion was located by navigation, we identified the central sulcus and dissected the arachnoid about 2 cm (Figures 3F–H). Then, the introducer and elliptic endoport were inserted under the guidance of the navigation pointer (Figure 3I). About 3.5 cm beneath the cortex, we saw the hemosiderin-stained surrounding parenchyma and hematoma (Figures 3J–L). The hematoma was evacuated and the lesion was dissected at the interface of the CCM and yellow-stained white matter. The CM resections were completed with special care to preserve the white matter tracts with the help of DTI navigation and intraoperative monitoring of evoked potentials (Figures 3M–P). After the endoport was removed, the cortex was intact without crush injury (Figure 3Q). One week later, conventional MRI was performed to confirm radical obliteration. The postoperative course was uneventful. On discharge, the muscle strength of the left upper limb and left lower limb was grade II and grade IV, respectively. Hypotonia, ataxia, and numbness of the left limbs were relieved. The Babinski sign was still positive on the left side. The pathological examination confirmed the diagnosis of CCM and hematoma (Figure 3S). Three months later, DTI and 3DT1 examination were performed again to compare with preoperative ones. The mean FA value of the ipsilateral CST was 0.45, and the fiber number was 139. The muscle strength was recovered to grade IV of the left limbs at 6 months after surgery (Figure 3T).

## Results

**Figure 4:**
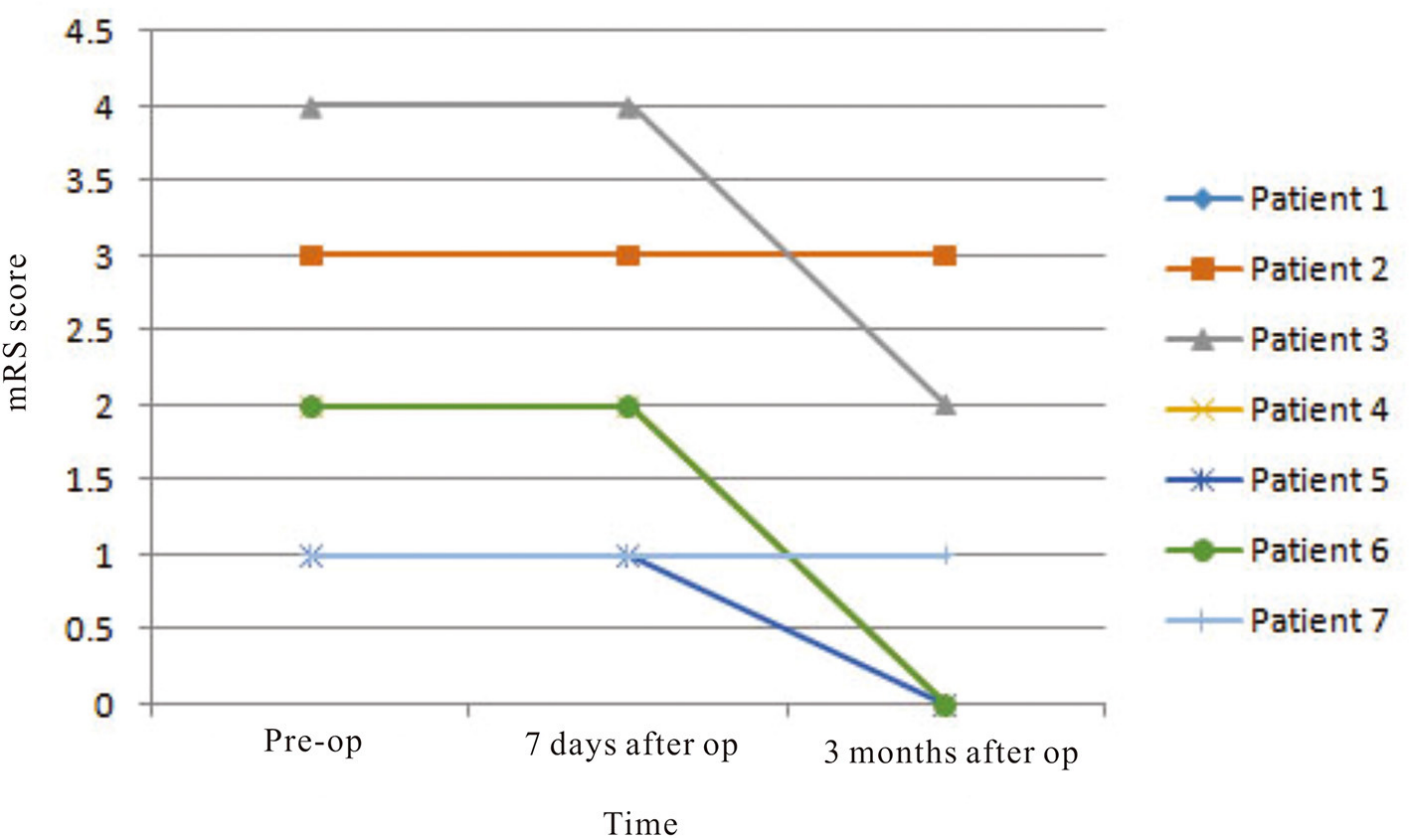
The mRS scores were improved significantly according to statistical analysis (*p* = 0.012).

Three male and four female patients with deep CCM and treated by endoscopic surgery were enrolled in this study, and their mean age at presentation was 49.0 ± 6.58 years old (Table 2). The mean size of the CCM and associated hematoma was 18.6 ± 12.40 mm. Two of the lesions were located at the corona radiata level and three at the basal ganglia level. According to preoperative physiological examination, five and three of the patients had muscle strength deficit of contralateral upper limbs and lower limbs, respectively. The preoperative mean value of mRS was 2.1 ± 1.07. One week after surgery, only two patients suffered from upper and lower limb muscle strength deficit. The mean value of mRS was 1.5 ± 0.98. Six months later, only one patient (14.3%) suffered from poor outcome (mRS = 3). In this patient, the preoperative muscle strength of the upper limb and lower limb was grade 3 and grade 4, respectively, which were not changed during follow-up. The mean mRS score recovered to 0.86 ± 1.22. The mRS scores were improved significantly according to statistical analysis (*p* = 0.012) (Figure 4).

**Table 2:** Variables associated with long-term motor deficits (Spearman nonparametric test).

**Variables**	**ULMS3 (*p*-value)**	**LLMS3 (*p*-value)**
Gender	0.211	0.203
Age	0.628	0.485
Location	0.672	0.513
Size	0.631	0.735
CSTFA1	0.264	0.282
NCST1	0.775	0.735
Follow-up	0.433	0.490
CSTFA2	0.259	0.277
NCST2	0.030	0.034

*ULMS3, muscle strength of contralateral upper limb at last follow-up; LLMS3, muscle strength of contralateral lower limb at last follow-up; CSTFA1, the overall mean FA values of ipsilateral CST on preoperative DTI; CSTFA2, the overall mean FA values of ipsilateral CST on postoperative DTI; NCST1, the number of fibers of ipsilateral CST on preoperative DTI; NCST2, the number of fibers of ipsilateral CST on follow-up DTI*.

**Figure 5:**
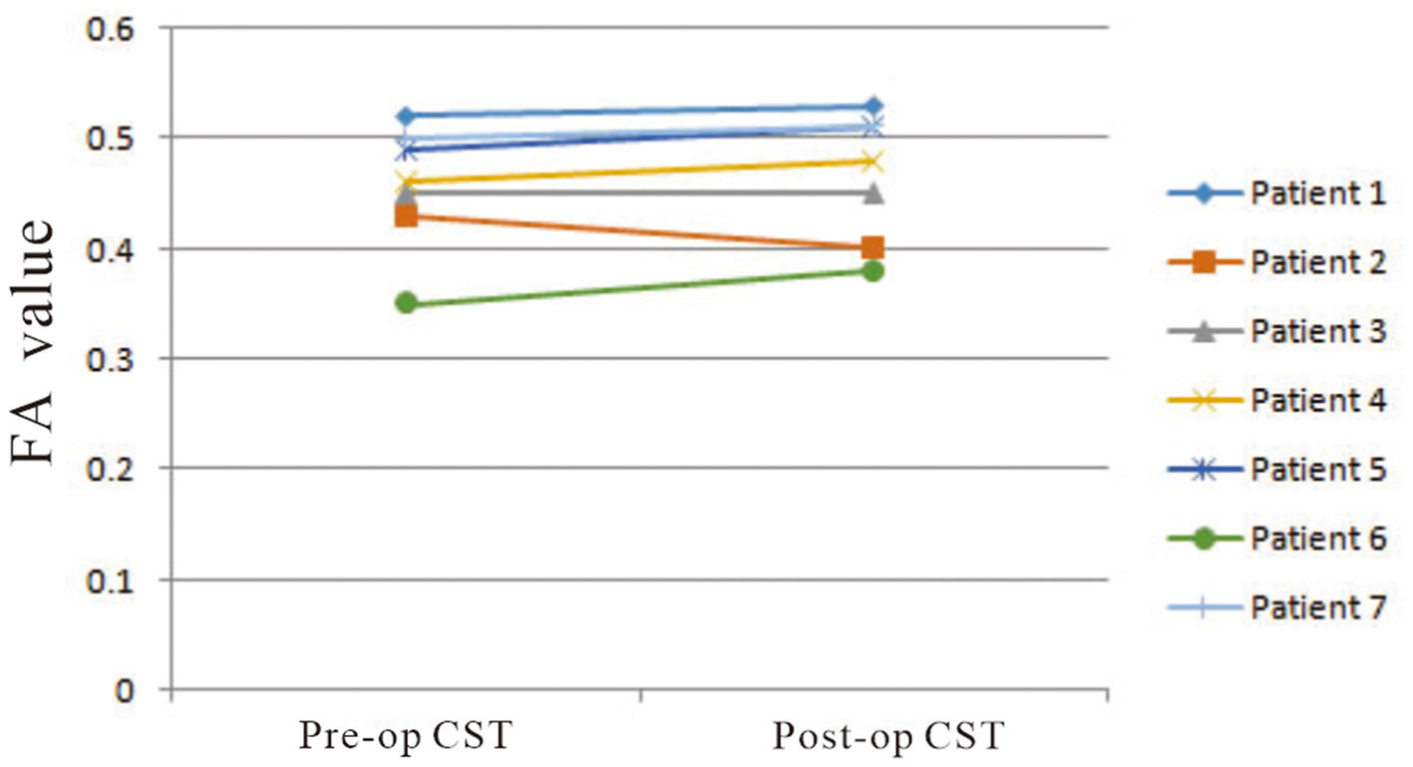
The change of mean FA values of ipsilateral CSTs on pre- and postoperative DTI images was not significant (*p* = 0.289).

**Figure 6:**
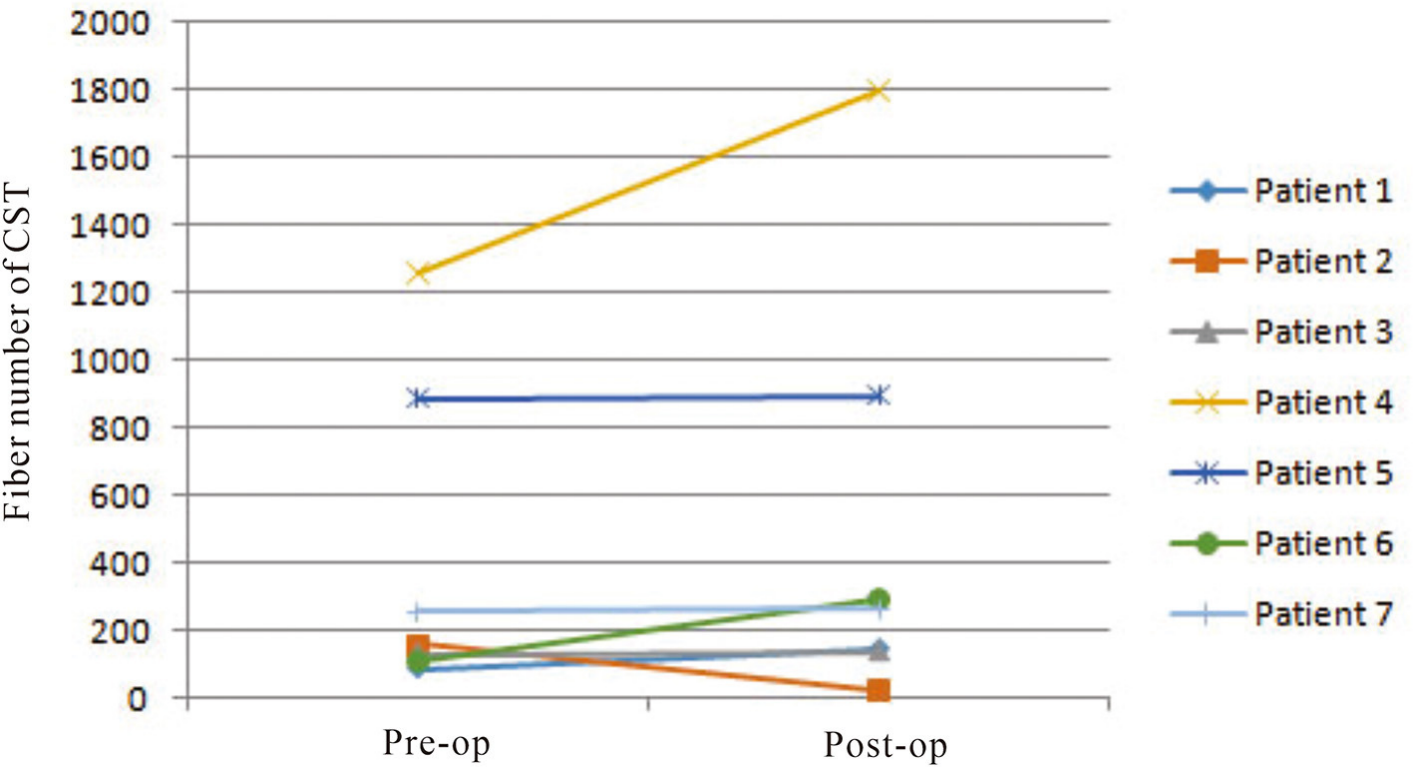
The change of fiber numbers of ipsilateral CSTs on pre- and postoperative DTI images was not significant (*p* = 0.289).

On preoperative DTI, the mean FA value of the ipsilateral CSTs was 0.46 ± 0.06, and the fiber number was 413 ± 465.88 (Table 3). Three months after surgery, on repeated DTI, the mean FA value of the ipsilateral CSTs was 0.47 ± 0.06, and the fiber number was 509.6 ± 636.4. According to the Spearman non-parametric test, only the fiber number of ipsilateral CST at 3 months after surgery was significantly associated with the muscle strength at last follow-up (more than 6 months) (*p* = 0.032). Compared with preoperative CST characteristics on DTI, the change of FA value (*p* = 0.289) and fiber number (*p* = 0.289) of ipsilateral CST on postoperative DTI was not significant (Figures 5, 6). It meant that the CST was spared during endoscopic surgery and recovered partly during 3 months interval. Only one patient, suffering from difficult hemostasis, had deterioration of FA value and fiber number of CST and poor outcome (mRS = 3).

**Table 3:** The difference of clinical outcomes and CST features between preoperative and at last follow-up.

**Variables**	**Preoperative mean value**	**At last follow-up mean value**	***P*-value**
mRS	2.14 ± 1.07	0.86 ± 1.22	0.012
CSTFA	0.46 ± 0.056	0.47 ± 0.058	0.289
NCST	413.29 ± 465.88	509.57 ± 636.45	0.289

*mRS, modified Rankin scale score; CSTFA, the overall mean FA values of ipsilateral CST; NCST, the number of fibers of ipsilateral CST*.

## Discussion

Deep location, long corridor of the surgical approach, distorted anatomical structures, and the indeterminate location of the fiber tracts make surgical resection of deep CCMs challenging. To facilitate safe surgery for supratentorial CMs in eloquent brain regions, Chang and colleagues proposed a system of 13 microsurgical approaches to six location targets with four general trajectories (8). In 2015, Rangel-Castilla and Spetzler conceptually divided the thalamus into six different regions to aid in the selection of the ideal surgical approach for a specific region: thalamic CCMs (9). However, those methods need extensive experience and surgical skills and only can be performed by senior surgeons. The application of DTI technology can clearly define the spatial relationship between CM and fiber bundles in white matter before surgery (10–12). A prospective randomized clinical trial conducted by us showed that DTI was able to non-invasively visualize fiber tracts *in vivo*, and the technique was valuable for complex neurosurgical planning to reduce morbidity when lesions involved the eloquent area (13). However, in our other study, after resection of supratentorial cavernous malformations adjacent to the corticospinal tract based on DTI navigation (≤10 mm), 37.5% patients had short-term surgery-related motor deficits, and 25.0% patients had long-term motor deficits at the last clinical visit (6). Most of the iatrogenic damage might be attributed to the injury of the CST fibers, which is caused by brain retractors and the difficult hemostasis. Thus, we concluded that the safe distance between CCM and CST was 3 mm to avoid CST damage. However, how to treat lesions in contact with the CST (<3 mm) is still challenging. Techniques allowing longer trajectories and access to deep CCMs without increased retraction of the surrounding brain tissue and direct visualization of the lesion and surrounding brain tissue are needed.

In 2019, Eichberg et al. reported a case series of 20 transcortical–transtubular approaches for resection of subcortical cavernomas with tubular retractor systems. They concluded that tubular retractors provide a low-profile, minimally invasive operative corridor for resection of subcortical cavernomas (14). However, they resected the lesions under microscopes. In this study, endoscopic surgery can expose the lesion with tubular retractors (endoports), and the view under endoscopy is panoramic and close and facilitates to identify the CCMs and hemosiderin-stained surrounding parenchyma. Combining the accuracy of DTI navigation and minimally invasive endoscopic surgery, it may be an effective way to remove supratentorial deep CCMs. In this study, seven patients were treated by endoscopic resection based on DTI navigation. Six months later, the mRS scores were improved significantly, and only one patient (14.3%) suffered from permanent muscle strength deficit. The morbidity was lower than the rate in our previous studies (25.0%) and that in the literature (10–30%). By comparing the CST characteristics on pre- and postoperative DTI, we found that most of the CST was protected during endoscopic surgery and recovered partly during follow-up. On preoperative DTI, the mean FA value of the ipsilateral CSTs was 0.46 ± 0.06, and the fiber number was 413 ± 465.88. Three months after surgery, on repeated DTI, the mean FA value of the ipsilateral CSTs was 0.47 ± 0.06, and the fiber number was 509.6 ± 636.4. That was the foundation for long-term good clinical outcomes. Several endoscopic technical improvements have been reported by different groups in the past years that enhanced orientation and safety (4). Suction and irrigation are applied to keep the surgical field clear. The key advantage of endoscopic surgery is a drastically reduced manipulation of surrounding brain tissue. Resection of CCM through conventional craniotomy often involves the use of brain retractors, and in order to visualize the bleeding sites, significant compression of surrounding brain tissue is often inevitable. Especially in deeply located ones, this is an important issue as more retraction on brain tissue is necessary for sufficient visualization. In contrast, endoscopic techniques allow longer access to deep CCMs and retraction of the surrounding brain tissue evenly and steadily (15, 16). Furthermore, the use of DTI neuronavigation easily facilitates access to the deep CCMs through considerable distances of non-eloquent brain tissue.

On the other hand, radiosurgery is also an alternative treatment for supratentorial deep cavernous malformation. A meta-analysis and review showed that deep cavernous malformations seem to benefit from radiosurgery for the reduction of annual hemorrhage rate in the first 2 and 2 years after (17). However, for patients with an enlarged lesion and progressed functional deficits, we preferred to perform a surgical resection to relieve mass effect and improve functions at a short term in our institute.

### Limitations

The major strength of this study is to provide an alternative surgical method to neurosurgeons. However, our study has several limitations: (a) because of the retrospective design of this study, general weaknesses and shortcomings are expected; (b) the sample size was small; and (c) this single center experience has only limited generalizability.

## Conclusion

Endoscopic surgery based on DTI navigation might be an effective method to protect fiber tracts in supratentorial deep CCM patient and improve long-term outcomes. However, more cases and studies are needed to confirm our findings.

## Data Availability Statement

The raw data supporting the conclusions of this article will be made available by the authors, without undue reservation.

## Ethics Statement

The studies involving human participants were reviewed and approved by the Institutional Review Board of The First Affiliated Hospital of Fujian Medical University. The patients/participants provided their written informed consent to participate in this study.

## Author Contributions

All authors listed have made substantial, direct and intellectual contribution to the work, and approved it for publication.

## Conflict of Interest

The authors declare that the research was conducted in the absence of any commercial or financial relationships that could be construed as a potential conflict of interest.
